# Recognition of depression by nurses in primary healthcare in Zimbabwe: Cross-sectional study

**DOI:** 10.1017/gmh.2026.10130

**Published:** 2026-01-22

**Authors:** Sakios Muduma, Malinda Kaiyo-Utete, Zoë Senter, Debra Machando, Kevin A. Hallgren, Emily C. Williams, Dixon Chibanda, Melanie Abas, Patience Mavunganidze, Graham Thornicroft, Helen E. Jack

**Affiliations:** 1 Department of Mental Health, Faculty of Medicine and Health Science, University of Zimbabwe, Zimbabwe; 2 Division of General Internal Medicine, University of Washington School of Medicine, USA; 3 World Health Organization Zimbabwe, Zimbabwe; 4 Department of Psychiatry and Behavioral Sciences, University of Washington School of Medicine, USA; 5 Department of Health Systems and Population Health, University of Washington, USA; 6 Department of Health Service and Population Health, Institute of Psychiatry, Psychology, and Neuroscience, King’s College London, UK; 7 Zimbabwe Ministry of Health and Child Care, Zimbabwe; 8 Centre for Global Mental Health, Institute of Psychiatry, Psychology, and Neuroscience, King’s College London, UK

**Keywords:** depression, primary care, mental health screening, implementation science

## Abstract

Depression is underrecognized in primary care, which is a barrier to treatment. For the last decade, Zimbabwe has invested in increasing access to depression treatment within primary healthcare. This study describes depression recognition by nurses and referral to treatment in four primary care clinics in Zimbabwe. Research staff screened 200 patients after they attended a primary care visit at a study clinic. They assessed depression using the PHQ-9 and assessed depression and/or anxiety using the Shona Symptoms Questionnaire (SSQ-14). Medical records were examined for depression and/or anxiety diagnoses. Positive depression and anxiety screens were compared with nurse documentation. 69.5% of participants were women and 56.5% were living with HIV. 6.0% had a PHQ-9 score ≥11, indicative of depression, and 22.0% had an SSQ score ≥9, indicative of depression and/or anxiety. None of the patients who screened positive for probable depression and/or anxiety were recognized by nurses. Nurses who saw the patients in the sample were surveyed. Most had not received formal training on mental health in primary care (mhGAP) prior to patient data collection. Despite efforts to expand depression treatment in Zimbabwe, individuals with probable depression were unrecognized by nurses, though nurses offered some care for other mental health conditions.

## Impact statement

Zimbabwe has been a focus of global investment in depression treatment and mental health services scale-up. The study findings, consistent with findings from many other African settings, demonstrated that Zimbabwean primary care nurses do not recognize depression when nurse recognition is compared with results on screening tools administered by a researcher. Nurses, however, documented providing social support to some patients and treating others for psychotic disorders, illustrating that they are integrating mental healthcare into their practice. We found that compared to patients without depression, many of the patients with probable depression presented with medication non-adherence or non-specific chronic pain, symptoms that could prompt a nurse to consider depression. However, many nurse participants had not had formal training in primary care–based management of depression, and they did not consider depression to be a high priority, relative to the other common conditions that they saw in their practice. We also observed that the primary care clinics are busy and understaffed, potentially leaving little time for depression management. These results highlight that depression recognition may be a barrier to treatment in primary care and, in addition, identify potential barriers to recognition. They also, however, suggest that nurses are addressing more severe mental illness in primary care, a potential success of mental health scale-up efforts.

## Introduction

Depression is common and often overlooked in primary healthcare (Patel et al., 2001; Menchetti et al., 2009). Many people with depression present to primary care with other concerns (Kapfhammer, 2006; Fekadu et al., 2022), and particularly in contexts where depression is stigmatized, patients may not bring up concerns about depression to their provider. As a result, depression can go unrecognized (Habtamu et al., 2023; Kohrt et al., 2025a), a barrier to treatment.

Several meta-analyses have examined depression recognition in primary care. One global meta-analysis found that among people with depression, based on a researcher-administered diagnostic test, 47.3% had their depression recognized (Egede, 2007; Fekadu et al., 2022; Habtamu et al., 2023; Mitchell et al., 2009). This study, however, included primarily studies from high-income countries and excluded nurses providing primary care. A meta-analysis in low- and middle-income countries (LMICs) found that 3.9% (range: 0–28%) of people with depression were recognized in primary care (Fekadu et al., 2022). Six of the nine studies in this systematic review included data from at least one African country (Ogunsemi et al., 2010; Kauye et al., 2014; Udedi, 2014; Fekadu et al., 2017; Ayinde et al., 2018; Rathod et al., 2018); of those, two found that primary care providers identified 0% of those with depression (Kauye et al., 2014; Udedi, 2014), and two found that they identified <2% (Fekadu et al., 2017; Ayinde et al., 2018). One study, which focused on physicians at a teaching hospital, found that the doctors identified 24.1% of people with depression (Ogunsemi et al., 2010), though this is not a standard primary care setting in much of Africa. Several interventions have been tested and shown to increase depression detection in African primary care, including a digital application to support primary care providers to identify and manage depression in Nigeria (Kohrt et al., 2025b), a 5-day provider training in Malawi (Kauye et al., 2014), provider training and implementation of routine screening in Ethiopia (Fekadu et al., 2025) and implementation of a broader collaborative care program that included depression screening in South Africa (Petersen et al., 2019). However, these practices are not routine in most LMIC primary care settings.

Over the past several decades, there has been a growing effort to improve treatment of people with depression in LMICs (Abas et al., 2003), with major investments from the World Health Organization (WHO) (WHO Special Initiative for Mental Health, n.d.), other international funders and national governments in depression treatment (Financing Mental Health, n.d.). Zimbabwe received international support to scale up the Friendship Bench, a lay health worker-delivered psychological therapy for depression and anxiety (Chibanda et al., 2016). There have been several efforts, led by both international organizations and the Ministry of Health, to train primary healthcare workers on care of mental health conditions, including depression, using the Mental Health Gap Action Programme Intervention Guide (mhGAP-IG) (WHO Special Initiative for Mental Health, n.d.). mhGAP-IG, however, does not include screening or focus on case recognition; rather, it starts at the step of differential diagnosis, once a provider has a clinical suspicion (World Health Organization, 2016).

In light of the ongoing investment in depression treatment in Zimbabwe, the aim of this study was to determine the proportion of people with probable depression who were identified and offered treatment in primary care. We hypothesized that the proportion of people who were recognized by the nurse is lower than the proportion of people who screened positive for depression. In order to better describe the broader context of mental healthcare delivery, we reviewed clinical notes for any examples of mental health treatment (Kohrt et al., 2025a). Understanding nurse practices in depression diagnosis and mental health treatment may inform ongoing efforts to implement mental health services within primary healthcare, both in Zimbabwe and in other LMICs.

## Methods

We used a cross-sectional study design to compare primary care nurse recognition of depression with the results of depression screening administered by a trained research assistant, similar to the design of depression recognition studies in other LMICs (Mitchell et al., 2009; Fekadu et al., 2017; Petersen et al., 2019; Kemp et al., 2020). This cross-sectional study is reported according to the STROBE Checklist (von Elm et al., 2007).

### Setting

In Harare, the capital of Zimbabwe (population 1.5 million), primary healthcare is provided by the City of Harare Health Department, delivered primarily by nurses and funded mainly through local taxes (City Of Harare | Council Health Facilities, n.d.; Abas et al., 2003). There are 41 public clinics: 29 small to medium clinics and 12 larger polyclinics (Shumba et al., 2025), which provide a wider range of services, including maternity care, laboratory services and occasional physician-delivered services. Based on our team’s knowledge of the healthcare system, patients presenting for primary care pay $USD 5 per visit, and those presenting for HIV care receive it for free. The clinics stock a limited supply of certain medications that nurses can prescribe. If medications are unavailable, patients must purchase them from private pharmacies.

Based on records from the Zimbabwean Ministry of Health, selected nurses in all Harare polyclinics had been exposed to the mhGAP training in the past 10 years and were supposed to be implementing mental health treatment per the mhGAP guidelines. Additional mhGAP training was going on during the study period. mhGAP trainings are typically delivered as one-time 5- to 9-day trainings, and they have been delivered by the Zimbabwean Ministry of Health, Medecins Sans Frontieres, and the WHO over the past decade (2014 to present). Primary healthcare staff from each clinic were selected to attend, with the idea that if some staff in the clinic are trained, they will pass their knowledge to the other clinic staff. The default for nurses in Zimbabwe is to use clinical judgment, not a screening tool, to assess for depression, which is consistent with WHO mhGAP-IG recommendations.

All polyclinics were implementing the Friendship Bench, which is delivered by lay health workers on a bench outside clinic buildings. Nurses can refer patients to the Friendship Bench. The lay health workers also recruit patients from the community to the Friendship Bench, typically people who approach them or who are referred by other community members.

Depression in Zimbabwe commonly presents with somatic symptoms (Patel et al., 2001) and in Zimbabwe’s primary language, Shona, there is not a word for depression. The closest idiom is *kufungisisa*, meaning “thinking too much” (Patel et al., 1997), but it does not capture the full range of depressive symptoms and can describe a normal, non-pathologic level of thinking.

### Sample and data collection

Patient data were collected between November 2023 and July 2024, and nurse data were collected between May and July 2024. Data collection occurred at four primary care polyclinics in Harare.

#### Patient sample

All people waiting for a primary care visit, including those presenting for HIV care, were handed a numbered card, and numbers were randomly selected. Those holding cards with the selected numbers were invited to participate. Individuals were excluded if they were pregnant, breastfeeding, not presenting for care (e.g., accompanying a family member), under age 18 or too acutely ill to participate. After agreeing to participate in the study, participants proceeded to their visit with the nurse.

A priori power analyses were conducted to inform the targeted sample size. We assumed that 40% (*n* = 80) of participants would have probable depression (Patel et al., 1997; Chibanda et al., 2016b, 2016a; Haas et al., 2023), with ≤20% (*n ≤*16) having depression recognized by their clinician (Fekadu et al., 2017; Petersen et al., 2019; Kemp et al., 2020). We found that a McNemar’s test would be well-powered (power ≥0.99) to detect a significantly lower rate of recognized depression compared to the rate of probable depression. Fifty patients were sampled from each of the four clinics.

#### Patient data collection

The Research Assistant (RA) gave the selected participants brief information about the study and screened them for eligibility. Prior to the nurse visit, participants were not informed that the study was about depression, so as not to bias them toward reporting depression to the nurse. As they would for any patient at the clinic, nurses documented their diagnosis and treatment plan in the patient’s medical record, a paper card that the patient brings to the clinic and carries home with them.

Following the nurse visit, patients reconvened with the RA, who went through the informed consent process, which included a detailed description of the study (though not its focus on depression), its risks and benefits and patient rights. If patients consented, the RA administered a demographic questionnaire and screened them for depression. The RA then reviewed the patient’s medical record for that day and recorded the notes word-for-word. The RA administered all questions verbally and recorded responses on a tablet. Patients were compensated $USD 10 in cash for participation.

Those who screened positive for moderate to severe depression or suicidality based on the PHQ-9 were referred to further care with a nurse-in-charge of the clinic.

#### Nurse sample

All nurses who saw any of the patient participants were invited to complete a survey (census sample). During interviews with patients, the RA noted down names and contact details for those nurses who attended to the patients participating in the study. Nurses who were not permanently assigned to a given clinic were excluded. Nurses were blinded to the study’s focus on depression so as not to bias them toward identifying depression.

#### Nurse data collection

After patient data collection at the clinic was complete, nurses were contacted and invited to participate in a survey. Nurses completed the survey directly on a tablet and were compensated $USD 10 to participate.

### Survey measures

#### Patient survey measures

In addition to a sociodemographic questionnaire that included a measure of food security (Food Security in the U.S. - Survey Tools | Economic Research Service, 2025), the RA used two tools to screen for mental health conditions:

1. *Patient Health Questionnaire-9 (PHQ-9):* The PHQ-9 is a 9-item questionnaire that is used worldwide to screen for depression (Kroenke et al., 2001). It asks questions about depressive symptoms in the past 2 weeks, and participants are asked to state how frequently they experience a symptom (not at all, several days, more than half the days, nearly every day). Scores range from 0 to 27. The PHQ-9 has been translated into Shona, Zimbabwe’s most common language, and validated against the Structured Clinical Interview of the DSM-IV diagnosis of depression with scores ≥11 indicating probable depression (sensitivity 85%, specificity 69%) (Chibanda et al., 2016b). In many settings, a PHQ-9 score of ≥10 is considered moderate to severe depression (Kroenke et al., 2001; Fekadu et al., 2017), and this score has been used in clinical trials in Zimbabwe (Abas et al., 2022). For this study, a cutoff of 11 was used for primary analyses, and sensitivity analyses were performed using a cutoff of 10.
2. *Shona Symptom Questionnaire (SSQ-14):* The SSQ-14 is a 14-item questionnaire that was developed in Zimbabwe to screen for common mental disorders, primarily depression or anxiety (Patel et al., 1997) and validated against the Structured Clinical Interview of the DSM-IV diagnoses of depression and/or generalized anxiety disorder (Chibanda et al., 2016b). The SSQ-14 asks 14 binary questions about symptoms experienced in the past week and is scored 0–14. Scores of ≥9 are considered positive.

As these are screening tools, not diagnostic instruments, we refer to a positive result as “probable depression” (PHQ-9) or “probable depression and/or anxiety” (SSQ-14).

Patients were asked why they presented to see the nurse that day and what treatment they received. Patients were also asked if they had ever been diagnosed with or treated for depression in the past and, if so, what treatment they received, excluding any treatment received that day.

#### Nurse survey measures

To characterize nurses, we used a questionnaire to collect sociodemographic characteristics, rank and details on prior mental health training, including WHO mhGAP training. To better understand why nurses may or may not identify and treat depression, the survey asked questions about their knowledge of mental health and the relative priority they assigned to depression in their practice. To assess knowledge, we administered the mhGAP knowledge test, a 24-question multiple choice test that is included in the training manuals for the WHO mhGAP-IG (score 0–24) (mhGAP Training Manuals, 2017). To assess clinical priorities, nurses were asked sequential sets of questions about the importance of various common health conditions in their practice, including depression. The lists of conditions were based on the Zimbabwe National Health Strategy 2021–2025 (Ministry of Health and Child Care, 2020).

### Data analysis

Descriptive statistics were used to characterize the patient and nurse samples and describe nurse scores on the mhGAP knowledge test. To display how nurses prioritized health conditions, the mean ranking was calculated for each medical condition and then the conditions were ordered by mean rank.

Medical record notes and patient-reported treatment plans were reviewed by three members of the research team and separately by a Zimbabwean nurse who was not part of the research team and was unaware of the research question. The notes were categorized as “depression recognized” or “depression not recognized,” and differences were reconciled through discussion.

Consistent with prior studies in LMICs (Petersen et al., 2019), we set very broad *a priori* criteria for identifying recognition of depression in the medical record: any mention of depression; referral to the mental health nurse or the Friendship Bench; prescription of an antidepressant; or mention of psychosocial problems, mental health issues, stress, grief or sadness. If a medication that can be used as an antidepressant was prescribed but another indication was noted, this was not considered recognition of depression (i.e., a tricyclic antidepressant that was noted to be used for sleep or pain).

The proportion of people with clinically recognized depression (medical record results) was compared with the proportion of people with probable depression based on the PHQ-9 using McNemar’s test. In a secondary analysis, probable depression or anxiety based on SSQ-14 scores was compared with medical record results using McNemar’s test.

We described symptoms and treatment plans from medical records for people with probable depression (PHQ-9 ≥ 11) and those with PHQ-9 scores of 0 to characterize how people with and without probable depression present to primary care.

## Results

### Patient sample

To reach a sample size of 200, 278 people were invited to participate. Fifty declined to participate, primarily because they were too busy, and 28 did not meet the inclusion criteria. Two participants were unable to complete the medical record review, as they were called back to see the nurse for additional follow-up care. They were included in the descriptive analyses and excluded from outcome analyses.

The patient sample was primarily female (69.5%). 56.5% self-reported living with HIV. 73% self-reported low to very low household food security. Patient sociodemographic characteristics are displayed in Table 1.

**Table 1.** Characteristics of the patient sample

Measure	Sample, n = 200 (%)
**Gender**	
*Man*	61 (30.5)
*Woman*	139 (69.5)
**Mean age (SD)**	41.6 (13.4)
**Level of education**	
*No formal education*	4 (2.0)
*Primary school*	32 (16.0)
*Secondary school*	136 (68.0)
*Advanced*	15 (7.5)
*Tertiary*	13 (6.5)
**HIV status (self-report)**	
*Negative*	82 (41.0)
*Positive*	113 (56.5)
*Unknown*	5 (2.5)
**Food security (USDA Household Food Security Survey Module)**	
*High food security (0)*	28 (14.0)
*Marginal food security (1–2)*	13 (7.5)
*Low food security (3–5)*	61 (30.5)
*Very low food security (6–10)*	85 (42.5)
**Employment status**	
*Unemployed*	63 (31.5)
*Self-employed*	97 (48.5)
*Formally employed in public sector*	11 (5.5)
*Formally employed in private sector*	29 (14.5)
**PHQ–9 score**	
*0–10 (no depression)*	188 (94.0)
*≥11 (depression based on Zimbabwean cut-point)*	12 (6.0)
*≥10 (depression based on international cut-point)*	20 (10.0)
**SSQ score (0–14)^a^**	
*0–8 (no depression and/or anxiety)*	156 (78.0)
*9–14 (depression and/or anxiety)*	44 (22.0)
**Self-report prior depression diagnosis^b^**	62 (31.0)
**Self-report prior depression treatment of those who self-report prior depression diagnosis (n = 62)**	
*Any treatment*	39 (62.9)
*Hypertension treatment*	11 (28.5)
*Counseling*	9 (23.1)
*Medication – patient does not remember which*	6 (15.4)
*Other medication^c^*	5 (12.8)
*Received treatment, but cannot remember what*	5 (12.8)
*Church*	2 (5.1)
*Amitriptyline*	1 (2.6)

a A single patient was missing a response to one of the 14 SSQ-14 questions, which was assumed to be negative; records were otherwise complete.

b Question was phrased “Have you ever been told that you have depression?”

c Other medications included pyridoxine, “sleeping tablets,” doxycycline, paracetamol and antihistamines.

#### Nurse identification of depression

Nurses identified two patients as having depression, one of whom was presenting for review of known depression. The other was presenting with joint pain, headache and insomnia and was given amitriptyline for 7 days, an NSAID, magnesium trisilicate and counseling; no specific diagnosis was written, but given that both counseling and amitriptyline were provided, depression was assumed, erring on the side of broader inclusion. Neither of these patients screened positive based on the SSQ-14 or PHQ-9.

#### Researcher identification of probable depression

Based on researcher-administered screening, 6% of the patient sample screened positive for probable depression using the PHQ-9 (cutoff of ≥11). If the international cutoff of ≥10 was used, 10% screened positive. Twenty-two percent screened positive for probable depression and/or anxiety using the SSQ-14.

#### Comparison of nurse and researcher identification

None of the patients who screened positive for depression based on the PHQ-9 with Zimbabwean cut-point or PHQ-9 with international cut-point, nor any of those who screened positive for depression and/or anxiety based on the SSQ-14 were identified by the nurse. The proportion of patients diagnosed with depression by the nurse was lower than the proportion of people who screened positive on the PHQ-9 with the Zimbabwean cut-point (score ≥11) (*χ*^2^ = 7.14 *p* < 0.01), the PHQ-9 with the international cut-point (score ≥10) (*χ*^2^ = 14.73, *p* < 0.01), or the SSQ-14 (*χ*^2^ = 37.36, *p* < 0.01).

Notably, reviewing medical notes, researchers disagreed on how to classify nurse recognition of two cases of probable depression. In both, medical notes described specific social challenges (i.e., “has risks at home” or “came late because had a child in hospital and also lost her aunt”), but the diagnoses and treatment were unrelated to mental health (i.e., prescribed HIV pre-exposure prophylaxis or the nurse did antiretroviral therapy adherence counseling). Through discussion, the study team decided that none were consistent with a depression diagnosis, as this captured life events and indicated no action toward diagnosis or treatment. The team also discussed several cases in which nurses provided antiretroviral therapy adherence counseling and concluded that this did not qualify as a diagnosis of depression, as adherence counseling is a distinct service that Zimbabwean polyclinics provide.

A summary of the reasons for presentation and treatment plan of patients with a PHQ-9 score ≥11 and those with a PHQ-9 score of 0 is displayed in Table 2.

**Table 2.** Reason for presentation and treatment plan for patients with low and high PHQ-9 scores. *Italicized* indicates a clinical presentation that could prompt clinical suspicion for depression

PHQ-9 score	Presentation	Treatment plan	Endorsed PHQ-9 #1 or #2	Endorsed PHQ-9 #9
18	*Non-adherence to antiretroviral treatment (ART), cough and returning sputum for TB testing*	Restart ART, health education on safer sex and chest X-ray	Yes	No
15	Blood pressure check and sore throat	Blood pressure medications and antibiotics	Yes	Yes
15	*Persistent headache, mildly elevated blood pressure*	Already on antibiotics; Refer to the tertiary care center for further management	Yes	Yes
15	*HIV testing and safety risks at home*	Tested HIV negative and prescribed PrEP	Yes	Yes
14	*Back pain and headache*	Prescribed diclofenac (NSAID) and antibiotics	Yes	Yes
13	Collect HIV medications	Given antiretroviral therapy	Yes	Yes
12	*Dizziness and malaise*	Told to increase intake of fluids and take 1 day off from work	Yes	Yes
12	Chest pain, cough and back pain	Prescribed antibiotics, increased fluid intake and paracetamol	Yes	Yes
11	*Back pain, leg pain*	Prescribed celecoxib and given one dose of diclofenac	Yes	Yes
11	*Numbness in left hand and pain in chest and left shoulder, rash*	Prescribed antibiotics and paracetamol	Yes	Yes
11	Collect HIV medications	Given antiretroviral therapy	Yes	Yes
11	Collect HIV medications	Given antiretroviral therapy	Yes	No
0	Refill HIV medications	Given antiretroviral therapy		
0	Refill HIV medications, cervical cancer screening	Given antiretroviral therapy, reviewed negative results of cervical cancer screening		
0	Productive cough and vomiting	Given antibiotics for pneumonia		
0	Productive cough, joint weakness, headache	Antibiotics and TB sputum testing		
0	Frequent, painful urination and vaginal itching	Prescribed antibiotics and painkillers		
0	Blood pressure check (blood pressure mildly elevated), intermittent headache	Urged to continue taking antihypertensives and given painkiller		
0	Refill HIV medications	Given antiretroviral therapy		
0	Refill HIV medications	Given antiretroviral therapy		
0	Refill HIV medications	Given antiretroviral therapy		
0	Refill HIV medications	Given antiretroviral therapy		
0	Refill HIV medications	Given antiretroviral therapy		
0	Breast lump	Probable breast cancer; referred to tertiary hospital for further management		
0	Refill HIV medications	Given antiretroviral therapy		
0	Chest pain and dyspnea	Prescribed antibiotics and 3 days off work		
0	Refill HIV medications	Given antiretroviral therapy		
0	Pus at recent surgical site	Prescribed antibiotics		
0	Long-acting antipsychotic injection	Given injection of long-acting antipsychotic (fluphenazine)		
0	Refill tuberculosis treatment	Given tuberculosis treatment		
0	Running nose	Prescribed antibiotics		
0	Abdominal pain, history of ulcers	Prescribed proton pump inhibitor, antibiotics and paracetamol		
0	Chest pain	Prescribed antibiotics and paracetamol; told to drink fluids and take time off work		
0	Refill HIV medications	Given antiretroviral therapy and tuberculosis medications		
0	Cough, fever	Prescribed antibiotics and TB sputum testing; asked to get HIV testing		
0	Dysuria, pain in genital area and lower abdominal pain	Prescribed antibiotics		
0	Refill HIV medications, cervical cancer screening	Given antiretroviral therapy, cervical cancer screening negative		

#### Patient-reported diagnosis and treatment

One patient with a PHQ-9 score of 10 (positive based on international cut-point) and SSQ-14 score of 9 (positive) reported that she was referred to counseling at a church and given sleeping pills. The nurse recorded that she was prescribed medication to prevent opportunistic infections and did not mention depression, counseling or sleeping pills. No other patients who screened positive based on either tool reported receiving a diagnosis of or treatment for depression that day.

Of the 62 people (31.0%) who reported that they had previously been diagnosed with depression, 62.9% reported that they previously received treatment, most commonly counseling (23.1%) or treatment with antihypertensive medications (28.5%).

#### Additional mental healthcare documented

Medical notes documented that the nurses provided some mental healthcare. As described above, one patient who screened negative on both PHQ-9 and SSQ-14 came for depression review, and the nurse refilled amitriptyline. Amitriptyline was also prescribed to several people for the management of chronic or neuropathic pain. Three people presented for review of a presumed psychotic disorder (notes did not specify a diagnosis). Two received a fluphenazine injection alone, and the other received refills of amitriptyline, haloperidol, chlorpromazine and benzhexol, and was given a fluphenazine injection. All screened negative on both the PHQ-9 and the SSQ-14.

### Nurse sample

Twenty-three nurses saw patients in the sample, of whom four were excluded due to not being permanently assigned to the clinic or not being able to be reached. Nineteen nurses participated in the survey (Table 3). Seventy-four percent of the nurse participants were women, with a mean age of 48 years. Three had most recently completed mhGAP training between 2015 and 2017, and five most recently completed mhGAP training in 2024, after patient data collection had taken place at their clinic.

**Table 3.** Characteristics of nurse sample

Measure	Sample, n = 19 (%)
**Gender**	
*Man*	5 (26.3%)
*Woman*	14 (73.7%)
**Mean age (SD)**	47.8 (4.8)
**Rank**	
*Registered general nurse*	8 (42.1%)
*OI/ART nurse*	1 (5.3%)
*Midwife*	5 (26.3%)
*Nursing sister*	2 (10.5%)
*Sister in Charge*	3 (15.8%)
**Mean number of years in practice (SD)**	19 (6.3)
**Received formal mhGAP training^a^**	9 (47.4%)
**Received informal or ongoing mhGAP training^b^**	5 (26.3%)
**Median mhGAP knowledge score out of 24 (IQR)**	
*Overall*	19 (16.5–22)
*mhGAP trained*	20.5 (19.75–23)
*Not mhGAP trained*	17 (15.5–20)

a Training took place in 2016, 2017 and 2024 and was delivered by Medecins Sans Frontiers, Ministry of Health and the World Health Organization. If nurses were trained in mhGAP more than once, the latest training date is listed here. Five nurses reported most recent mhGAP training dates that occurred after patient data collection had begun at their clinic.

b Only nurses who reported receiving formal mhGAP training reported receiving any informal or ongoing mhGAP training.

The median score on the mhGAP knowledge test was 19/24 for all nurses, 20.5/24 for those who had previously been trained in mhGAP, and 17/24 for those who had not previously been trained. Prioritization of medical conditions within their practice is displayed in Table 4. Communicable diseases were a higher priority than non-communicable diseases or reproductive, maternal and child health. Among non-communicable diseases, mental health was ranked third of seven, and depression was the highest priority mental health condition.

**Table 4.** Nurse priorities within clinical practice (Ministry of Health and Child Care, 2020)

Health condition	Mean rank	Overall rank
**What do you think is the most important in your clinical practice?**		
Communicable diseases	1.74	1
Reproductive, maternal and child health	1.84	2
Non-communicable diseases	2.42	3
**Among non-communicable diseases, what do you think is most important in your clinical practice?**		
Hypertension and cardiovascular health	2.16	1
Diabetes	3.00	2
Mental health^a^	3.50	3
Injuries	4.11	4
Cancer	4.58	5
Oral health	4.95	6
Rehabilitation	5.53	7
**Among commonly seen health conditions, what do you think is most important in your clinical practice?**		
Maternal health	2.42	1
HIV	2.58	2
Tuberculosis	3.11	3
Hypertension	3.68	4
Depression	3.74	5
Malaria	5.47	6
**Among mental health conditions, what do you think is most important in your clinical practice?**		
Depression	2.42	1
Psychoses	3.11	2
Child and adolescent mental health	3.63	3
Substance use disorders	3.68	4
Epilepsy	3.84	5
Self-harm and suicide	5.11	6
Dementia	6.21	7

a Missing one response.

## Discussion

We assessed depression recognition by primary care nurses in the context of a decade of ongoing efforts to scale up mental healthcare in primary healthcare in Zimbabwe. We found that nurses did not record diagnosis or treatment of depression for any of those who screened positive for depression based on either the PHQ-9 or the SSQ-14. Prevalence of depression in the sample, however, was lower than anticipated, and most of the nurses had never received formal mhGAP training. Based on review of medical notes and patient-reported treatment plans, we identified several instances in which mental healthcare was documented, including referral to counseling at a church and refills of existing psychotropic medications, which suggests an important success of mental health integration into primary care.

There are several reasons why nurses may not have recognized people with probable depression. First, primary care nurses face many competing demands and have limited staffing. Many nurses have left Zimbabwe due to poor working conditions and inadequate salaries, leaving unfilled roles in primary care. There were both cholera and dysentery outbreaks during the study period, which pulled staff away from the clinic. Lack of time is cited as a barrier to depression recognition in studies in both LMICs and high-income countries (Cepoiu et al., 2008; Zimba et al., 2025), and nurses in Zimbabwe face extreme time constraints, with about 55% of nurses taking less than 10 minutes with each patient (Chikanda, 2005). Due to the lack of time, nurses likely had to prioritize what issues they addressed for each patient. Even if they had the clinical skills to recognize depression, they may have chosen not to due to competing priorities. The nurses in this study reported that depression is not a high priority, ranking it fifth of six in terms of importance among commonly seen conditions in primary care, below maternal health, HIV, tuberculosis and hypertension, all of which were very common based on our review of medical records. Additionally, nurses may not have had adequate training on how to identify depression. Very few nurses had been formally trained in mhGAP prior to patient data collection, even though all clinics had been included in prior trainings. The low proportion of nurses trained is likely due to high nurse turnover in public sector clinics. Finally, nurses may have normalized depressive symptoms in the challenging context in which they work: the African cultural idiom “Upenyu mutoro,” translated to “life is a burden,” frames *kufungisisa* (thinking too much or depression) as normal, potentially causing nurses to accept depressive symptoms as the norm amidst the general challenges many of their patients face (Ngwenya, 2020).

There was, however, clear evidence that nurses were incorporating mental health into their practice. Three patients received refills of medications for a presumed psychotic disorder, and another received an antidepressant refill and had recovered from depression based on screening results. Nearly one-third of patient participants reported having previously been diagnosed with depression, and 23.1% of those reported having received counseling. The plurality of those with prior treatment (28.5%), however, stated that they had received treatment for hypertension. This is consistent with prior research in Zimbabwe showing that depression and hypertension are seen as synonymous (Kidia et al., 2024), both as manifestations of stress. This makes it challenging to interpret what a prior diagnosis of depression means to patient participants, and it is likely not all were diagnosed with depression as understood in a Western medicine context.

Examination of the presentations of patients with PHQ-9 scores consistent with probable depression shows that many people presented with chronic, non-specific pain or dizziness or with medication non-adherence. None of the patients with PHQ-9 scores of 0 presented this way. Depression would conceivably be on the differential diagnosis for patients with these presentations, given that somatic manifestations of depression (Caballero et al., 2008) are common in Zimbabwe, and HIV medication non-adherence has also been associated with depression in several prior studies (Mayston et al., 2012; Nakimuli-Mpungu et al., 2012).

The prevalence of depression in this study was lower than we anticipated. Other studies in Zimbabwean primary care have found very high prevalence of depression (up to 70%) (Chibanda et al., 2016b; Verhey et al., 2018; Velloza et al., 2021), markedly higher than many global and regional estimates (GBD, 2019; Petersen et al., 2019). Within Zimbabwe, prevalence estimates in primary care, HIV care and community settings have varied considerably (Mebrahtu et al., 2020), with many in community settings and several in HIV or TB care finding a prevalence of <20% (Abas and Broadhead, 1997; Sebit et al., 2002; Haney et al., 2014; Chin et al., 2019; Machisa and Shamu, 2022). Variation in whether a screening or diagnostic tool was used and what tool was used may explain some of these differences. The prevalence that we found – 6% prevalence of probable depression with PHQ-9 and 22% prevalence of probable depression or anxiety with SSQ-14 – aligns with the lower end of Zimbabwean estimates (Patel et al., 1997; Sebit et al., 2003; Chibanda et al., 2016b; Haas et al., 2023) and most global and regional estimates (GBD, 2019).

These findings have several implications for practitioners and policymakers. First, routine screening for depression is one strategy for improving depression detection, and screening has been used successfully in a limited number of other African settings, often when tied with broader efforts to support mental health treatment within primary care (Petersen et al., 2019; Fekadu et al., 2025). Since this study was conducted, the Zimbabwean Ministry of Health has begun encouraging universal screening with the PHQ-9. However, it is not yet clear if screening is acceptable, feasible or appropriate in Zimbabwe (Oladeji et al., 2015; Reynolds and Patel, 2017; Fekadu et al., 2025). Second, neither nurses who had been trained in mhGAP nor those who had not been trained in mhGAP recognized depression, and both groups had relatively high scores on the mhGAP knowledge test. This suggests that many nurses may have the clinical knowledge of how to treat depression, but they do not know how to identify it or do not prioritize identifying it within their practice. Future interventions could incorporate greater emphasis on case recognition into both training and ongoing supervision, and highlight common presenting symptoms in the Zimbabwean cultural context for which depression should be on the differential diagnosis, such as chronic pain, headaches or medication non-adherence (Table 2). To prioritize depression in the resource-limited Zimbabwean context, nurses may need more well-staffed clinics and additional support to address the many other urgent priorities they see. Finally, we found a very high prevalence of food insecurity and informal employment or unemployment. Particularly in light of the challenging economic and political situation in Zimbabwe, the bidirectional relationship between poverty and depression should be considered in efforts to scale up depression screening and treatment (Lund and Cois, 2018; Ridley et al., 2020; Califf et al., 2022; Lund et al., 2023).

This study has several limitations. First, we used screening tools, not diagnostic interviews, to determine cases of probable depression, a feature shared by most studies of depression identification in Africa (Fekadu et al., 2022; Kohrt et al., 2025a). However, we chose a higher cut-point (11) to reduce the risk of false positives and conducted additional analyses using a locally developed, cross-diagnostic tool (SSQ-14). Second, medical notes may not capture everything that happened in a visit, particularly in a busy primary care clinic and for a highly stigmatized condition. Nurses may have provided counseling or treatment for depression without recording it in the medical record (Mitchell et al., 2009), perhaps due to stigma. Third, we cannot draw any conclusions about a specific mhGAP training or about mhGAP broadly, particularly as most nurses had not been exposed to formal mhGAP training, and repeat training was ongoing. Rather, we examined current practices in a real-world environment that has been exposed to multiple efforts to improve mental healthcare. Fourth, we sampled a relatively small number of patients with depression across four primary care clinics. Depression recognition may differ in other parts of the country. Fifth, the nurse survey used international, standardized tools to assess mental health knowledge, so it was not able to assess local idioms or understandings of depression and used the word “depression” rather than the local language word *kufungisisa.*

## Conclusion

Despite multiple efforts to increase depression treatment in Zimbabwe, nurses documented no recognized cases of depression among those who screened positive based on a researcher-administered screening tool. In Zimbabwe, recognition is a barrier to depression treatment. Routine screening in primary care or additional focus on depression diagnosis and management in primary care during medical and nursing school may help increase recognition. Future research should examine the acceptability, feasibility and effectiveness of different types of nurse training on depression recognition and management, and should examine strategies for patient screening, including self-administered screening, targeted screening for high-risk groups and routine screening for all patients.
