# Supplementary material for: Recognition of depression by nurses in primary healthcare in Zimbabwe: Cross-sectional study
Source: Glob Ment Health (Camb). 2026 Jan 22;13:e30. doi: 10.1017/gmh.2026.10130 (PMC12936446; doi:10.1017/gmh.2026.10130)
Supplement: Peer review history

# Reviewer report: Review: Recognition of depression by nurses in primary healthcare in Zimbabwe: Cross-sectional study — R1/PR2

**DOI:** 10.1017/gmh.2026.10130.pr2

**Authors:** Charlotte Bernard

**Affiliations:** University of Bordeaux Department of Life and Health Sciences, France

Authors addressed my concerns. I recommand the publication of their manuscript

# Reviewer report: Review: Recognition of depression by nurses in primary healthcare in Zimbabwe: Cross-sectional study — R1/PR3

**DOI:** 10.1017/gmh.2026.10130.pr3

**Authors:** Kassahun Habtamu

The authors addressed all of the comments. I have no any further comments.

# Editor report: Recommendation: Recognition of depression by nurses in primary healthcare in Zimbabwe: Cross-sectional study — R1/PR4

**DOI:** 10.1017/gmh.2026.10130.pr4

**Affiliations:** University of Zimbabwe Faculty of Medicine, Zimbabwe

No accompanying comment.

# Editor report: Decision: Recognition of depression by nurses in primary healthcare in Zimbabwe: Cross-sectional study — R1/PR5

**DOI:** 10.1017/gmh.2026.10130.pr5

**Affiliations:** Johns Hopkins University Bloomberg School of Public Health, United States

No accompanying comment.
